# Protective Effects of Magnolol, Rutin, and Gallic Acid in Broilers Challenged with Dietary Oxidized Soybean Oil

**DOI:** 10.3390/antiox14101186

**Published:** 2025-09-28

**Authors:** Fang Chen, Feng Jin, Encun Du, Wenjing Tao, Na Zhao, Qiwen Fan, Jintao Wei

**Affiliations:** 1 Institute of Animal Husbandry and Veterinary Sciences, Hubei Academy of Agricultural Sciences, Wuhan 430064, China; gwz949@hbaas.com (F.J.); duencun@hbaas.com (E.D.); taowenjing1127@hbaas.com (W.T.); hnzona@hbaas.com (N.Z.); qwfan@hbaas.com (Q.F.); 2 Hubei Key Laboratory of Animal Embryo and Molecular Breeding, Wuhan 430064, China; 3 Key Laboratory of Prevention and Control Agents for Animal Bacteriosis, Wuhan 430064, China

**Keywords:** antioxidant capacity, hepatic lipid metabolism, intestinal barrier, microbiota, metabolome

## Abstract

This study evaluated the protective effects of magnolol, rutin, and gallic acid in broilers fed oxidized soybean oil. Four hundred seven-day-old male Arbor Acre broilers were randomly assigned to five treatments with eight replicates each: CON (4% fresh oil), OOC (4% oxidized oil), and OOC supplemented with 200 mg/kg of magnolol (MAG), rutin (RUT), or gallic acid (GAA). OOC significantly reduced 42-day body weight (BW), average daily gain (ADG), and average daily feed intake (ADFI), reduced serum antioxidant enzyme activities (T-SOD, GSH-Px) and elevated malondialdehyde and triglyceride levels. It also upregulated hepatic lipogenic (*FASN*, *ACACA*, *SREBP-1*) and inflammation (*NF-κB1/2*) genes, damaged intestinal morphology, reduced cecal *Erysipelatoclostridium* and *Shuttleworthia* abundances, and elevated oxidized lipids (9,10-DiHOME and prostaglandin G2) in breast muscle. All three polyphenols increased ADFI (22–42 d), ileal villus height and *ZO-1* expression, while reducing serum triglycerides, ileal MDA, and hepatic *NF-κB2* expression. Both magnolol and rutin further enhanced BW (42 d) and ADG (7–42 d), decreased *ACACA* expression, and elevated cecal *Lachnoclostridium* abundance. Additionally, magnolol significantly decreased the contents of 9,10-DiHOME and malondialdehyde, while rutin reduced prostaglandin G2 levels in the breast muscle. In conclusion, polyphenol supplementation alleviated oxidized oil-induced adverse effects, with magnolol and rutin being more effective.

## 1. Introduction

Oil is an essential component of poultry diets, providing energy, facilitating the absorption of fat-soluble nutrients, enhancing palatability, reducing feed dust [1], and improving heat tolerance in chickens [2]. However, the high concentrations of unsaturated fatty acids in vegetable oils renders them highly susceptible to oxidation, particularly in hot climates or during feed processing. Oxidized oils exhibit not only compromised nutritional value but also accumulate a range of lipid oxidation products [3] that may cause direct harm to animal health. Studies demonstrated that oxidized oils induce oxidative stress, compromise immune function, and trigger inflammatory response [4,5]. Additionally, oxidized oils can impair liver function, disrupt lipid metabolism, and promote lipid deposition [6,7]. Oxidized oils also compromise intestinal barrier integrity, and disrupt the intestinal microbiota [8,9]. Furthermore, oxidized oils increase susceptibility to oxidation in meat and elevate lipid hydroperoxide content [10]. Therefore, it is crucial not only to prevent oil oxidation in feed but also to adopt effective nutritional strategies, such as adding dietary additives, to mitigate the negative effects of oxidized oils on broiler health and meat quality.

Polyphenols are known for their antioxidant, anti-inflammatory, and hepatoprotective properties [11]. Among diverse natural polyphenols, magnolol, rutin, and gallic acid have garnered attention due to their strong antioxidant properties and multiple health benefits. Magnolol is a bioactive compound form *Magnolia officinalis*. Rutin and gallic acid, are widely distributed in fruits and vegetables. The potential as feed additives of these three polyphenols have been extensively studied. Xie et al. reported that dietary supplementation with 200 and 300 mg/kg improved growth performance, meat quality of the broilers and antioxidant capacity, and modulated gut microbiota homeostasis [12]. Our previous studies have also confirmed that the addition of 200–300 mg/kg magnolol improved the antioxidant capacity and intestinal health of laying hens [13]. Li et al. reported that dietary adding 200 and 400 mg rutin improved meat quality and the antioxidant capacity of Qingyuan partridge chickens [14]. Xiong et al. reported that dietary addition of 150–450 mg/kg gallic acid alleviated the effect of the stress response on the growth performance of broiler chickens and improve antioxidant capacity and meat quality [15]. Although the health-promoting effects of these polyphenols in animals have been widely documented, their protective efficacy against oxidized oil-induced damage have not been sufficiently investigated. Moreover, although all three compounds belong to the polyphenol class and share certain fundamental biological properties, they originate from different subclasses. These fundamental structural differences may lead to significant variations in their specific physiological functions. Therefore, this study evaluated the effects of magnolol, rutin, and gallic acid on growth performance, serum biochemistry, antioxidant status, hepatic lipid metabolism, intestinal barrier function, gut microbiota, meat quality, and muscle metabolome in broilers challenged with dietary oxidized soybean oil. This study will provide a scientific basis for their potential application in poultry production.

## 2. Materials and Methods

### 2.1. Preparation of Oxidized Oil

Fresh soybean oil (Fulinmen, COFCO Corporation, Beijing, China) was purchased from local supermarkets. The oxidized oils were prepared as previously described [16]. Briefly, the oil was placed in an uncovered container and exposed to outdoor sunlight at temperatures ranging from 30 to 40 °C for 8 h per day over a period of 60 days (from July to August). The same batch of fresh oil was stored in a cool, dark environment until use. Peroxide values of oil and diet were analyzed using the iodometric titration method according to the China National Industry Standard NY/T 4424-2023 [17]. The peroxide value of the oxidized oil was 41.90 mmol/kg, compared to 1.82 mmol/kg for the fresh oil.

### 2.2. Experimental Design, Diets, and Management

The animal trial was conducted at the Animal Nutrition Research Facility of the Institute of Animal Science, Hubei Academy of Agricultural Sciences between May to June 2024. All experimental procedures involving animals were conducted according to the guidelines in the Laboratory Animals-General Code of Animal Welfare (GB/T 42011-2022, China).

One-day-old male Arbor Acre chicks were purchased from Hubei Zhengda Co., Ltd. (Shuizhou, China). After a week of acclimatization, a total of four hundred broilers aged 7 days with similar body weight (150 ± 3 g) were randomly allocated to five treatments (8 replicate cages per group, 10 birds per cage) using a completely randomized design: a fresh oil group (CON), an oxidized oil group (OOC), and oxidized oil diet supplemented with 200 mg/kg magnolol (MAG), rutin (RUT), or gallic acid (GAA), respectively. The peroxide value of oxidized oil diet was measured at 2.40 mmol/kg, compared to 0.22 mmol/kg for the diet of the CON group. Magnolol, rutin, and gallic acid (all with purity > 98%) were purchased from ConBon Biotech Co., Ltd. (Chengdu, China). The basal diet was a standard maize/soybean meal diet in mash form. Diets for starter (1 to 21 d) and grower phases (22 to 42 d) are presented in Table 1. Experimental diets were produced by adding 4% fresh or oxidized soybean oil. Each replicate of chicks was housed in a 1 m × 1 m flat-line cage equipped with a feeder and an automatic nipple drinker. Broilers were maintained at 34 to 35 °C for the first three days, after which the temperature was reduced by approximately 1.5 °C every three days until it reached 24 ± 1 °C by day 21. Relative humidity was maintained at 55–65% throughout the trial. All chicks had free access to clean water and feed.

### 2.3. Sample Collection

Feed supplied and residues per replicate were recorded weekly for feed conversion ratio (FCR) and average daily feed intake (ADFI) calculation. Body weight (BW) was recorded on d 21 and 42, with average daily gain (ADG) subsequently calculated. On day 42, one healthy broiler with a body weight close to the replicate average was selected from each replicate. Following a 12 h fast, a blood sample was obtained from the veins of the birds’ wings. After 2 h, the blood was centrifuged at 3000× *g* at 4 °C for 10 min to obtain serum. Then, the birds were slaughtered via carbon dioxide asphyxiation followed by exsanguination to ensure death, and samples were collected by dissection. Distal segments (1 cm) of jejunum and ileum were collected and fixed in 10% neutral buffered formalin for histology. After longitudinal incision and content removal, mucosal scrapings were obtained from jejunum and ileum using sterile glass slides. Samples of jejunum, ileum, liver tissue, pectoral muscle and cecal content were flash-frozen in liquid nitrogen and stored at −80 °C.

### 2.4. Slaughter Performance and Organ Index

The gizzard (without the cuticle), heart, thymus, liver, spleen, pancreas, and bursa of Fabricius of birds were collected and weighed. The relative weights (weight of organ/BW × 100%) were calculated. Slaughter performance, including carcass yield, half-eviscerated rate, eviscerated rate, thigh muscle rate, breast muscle rate, and abdominal fat rate were measured according to the China National Industry Standard NY/T 823-2020 [18].

### 2.5. Analyses of Serum Biochemical Indices

Aspartate amino transferase (AST), alanine aminotransferase (ALT), total protein (TP), albumin (ALB), glucose (GLU), uric acid (UA), urea, lactate dehydrogenase (LDH), high-density lipoprotein cholesterol (HDL-C), low-density lipoprotein cholesterol (LDL-C), total cholesterol (TC) and triglyceride (TG) were measured using the colorimetric method (UV-2550, Shimadzu, Japan) with commercial kit (C010, C009, A045, A028, F006, C012, A020, A112, A113, A111, A110, Nanjing Jiancheng Bioengineering Institute, Nanjing, China).

### 2.6. Antioxidant Status Assay

Mucosal samples (jejunum, ileum) and liver tissue (*n* = 8 per group) were immediately weighed, homogenized in ice-cold PBS (1:9, *w*/*v*), and centrifuged (4000× *g*, 4 °C, 10 min). Supernatants were collected for analysis. Total superoxide dismutase (T-SOD) and glutathione peroxidase (GSH-Px) activities, along with malondialdehyde (MDA) content, were determined in serum and tissue homogenates using the colorimetric method (UV-2550, Shimadzu, Japan) with commercial assay kits (A001, A005, and A003, Nanjing Jiancheng Bioengineering Institute, Nanjing, China). Results of the tissue were normalized to protein concentration, quantified by the BCA Protein Assay Kit (A045-2, Nanjing Jiancheng Bioengineering Institute, Nanjing, China).

### 2.7. Histological Studies

After fixation in formalin solution for 24 h, jejunum and ileum tissues (n = 8) were paraffin-embedded and sectioned at 4 μm thickness. Sections were stained with hematoxylin and eosin for histomorphometry analysis. Eight intact villi per section were randomly selected to determine villus height (VH), crypt depth (CD), and bowel wall thickness (BWT).

### 2.8. RNA Extraction and Real-Time PCR

Total RNA was extracted from jejunum, ileum, and liver tissues using TRIzol™ reagent (Takara, Dalian, China). cDNA synthesis was performed with the PrimeScript™ RT reagent kit (Takara), followed by qRT-PCR using SYBR^®^ Premix Ex Taq™ (Takara, Dalian, China) on an Applied Biosystems 7900HT system (Foster City, CA, USA). All reactions were run in triplicate on 384-well plates. mRNA levels were normalized to β-actin expression, with relative gene quantification calculated via the 2^−ΔΔCt^ method. The primer sequences are listed in Table S1.

### 2.9. Meat Quality Analysis

The right breast muscle was collected intact for meat quality analysis. After 24 h storage at 4 °C, pH was determined using a digital pH meter. Meat color parameters, L* (lightness), a* (redness), and b* (yellowness) were measured using a chromameter (CR-10 Plus, Konica Minolta Optics Co., Ltd., Tokyo, Japan). Three random readings were taken from different locations on the meat surface and averaged. Shear force (N/cm^2^) was determined using a texture analyzer on three 0.5 cm diameter muscle fiber-parallel cores per sample. For drip loss assessment, 2 × 2 × 1 cm breast samples (m_1_) were sealed in tubes, stored at 4 °C for 24 h, and reweighed (m_2_). Drip loss (%) was calculated as (m_1_ − m_2_)/m_1_ × 100.

### 2.10. Quasi-Targeted Metabolomics Analysis

Breast tissue samples (0.1 g) were individually pulverized in liquid nitrogen and homogenized in pre-cooled 80% methanol. After 5 min ice incubation, homogenates were centrifuged (15,000× *g*, 4 °C, 20 min). An aliquot of supernatant was diluted with LC-MS grade water to 53% methanol concentration, re-centrifuged (15,000× *g*, 4 °C, 20 min), and the final supernatant was injected into the UHPLC-MS/MS system. UHPLC-MS/MS analyses were performed using a Vanquish UHPLC system (ThermoFisher, Pittsburgh, Germany) coupled with an Orbitrap Q Exactive^TM^ HF mass spectrometer at Novogene Co., Ltd. (Beijing, China).

Raw data files were processed with Compound Discoverer 3.3 (Thermo Fisher, Pittsburgh, Germany) for peak alignment, peak picking, and metabolite quantification. Background ions were removed using blank samples. Peak intensities were normalized to the total spectral intensity. The aligned peaks were matched against the mzCloud (https://www.mzcloud.org/, accessed on 8 August 2024), mzVault, and MassList databases for accurate metabolite identification and relative quantification. Metabolites were annotated against KEGG (https://www.genome.jp/kegg/pathway.html, accessed on 8 March 2024), HMDB (https://hmdb.ca/metabolites, accessed on 8 August 2024), and Lipidmaps (http://www.lipidmaps.org/, accessed on 8 August 2024) databases. Statistical analyses were performed using the statistical software R (R versionR-3.4.3). When data were not normally distributed, normal transformations were attempted using of area normalization method. Significantly differentially expressed metabolites (DEMs) were identified based on variable important in projection (VIP) values from the OPLS-DA model and *p*-values from Student’s *t*-test (VIP > 1, *p* < 0.05, |Fold change| ≥ 1.5). The bubble plot of DEMs was generated using the ggplot2 package in R. The KEGG database was used to investigate the functions and metabolic pathways of metabolites. A metabolic pathway was considered significantly enriched when its *p*-value < 0.05.

### 2.11. 16S rDNA Gene Sequencing of the Cecal Microbiome

Microbial genomic DNA from cecal content samples (meeting purity/concentration thresholds) was used to amplify the V3–V4 hypervariable regions of 16S rDNA with primers 341F and 806R (Table S1). Indexed adapters were added to the ends of the 16S rDNA amplicons to create indexed libraries for sequencing on an Illumina NovaSeq 6000 platform (Illumina, San Diego, CA, USA), performed by Novogene Co., Ltd. (Beijing, China). The obtained sequences were then aligned into operational taxonomic units (OTUs) based on 97% sequence similarity. Alpha diversity indices, including Shannon, Simpson, Chao1, and Dominance were calculated using QIIME (Version 1.9.1). Principal coordinate analysis (PCoA) based on unweighted UniFrac distance metrics was conducted to visualize beta diversity. Representative OTU sequences were taxonomically classified using the RDP Classifier against the Silva_132 16S rRNA database (http://www.arb-silva.de/, accessed on 10 August 2024) at an 80% confidence threshold across multiple taxonomic levels, including kingdom, phylum, class, order, family, and genus. Differences between groups were assessed using the Wilcoxon test. Predictive functional profiles of the microbial communities were inferred from 16S rRNA marker gene sequences with Tax4Fun, leveraging KEGG orthology predictions derived from the KEGG pathway database. Spearman’s rank correlations analyses were performed using R software (R Foundation for Statistical Computing, Vienna, Austria).

### 2.12. Statistical Analyses

Experimental data on growth performance, immune organ index, serum biochemical index, intestinal histomorphology, meat quality, and the relative quantification of genes were analyzed using one-way analysis of variance (ANOVA) in SPSS (version 20.0; IBM Inc., Armonk, NY, USA). Prior to analysis, the normality of the data and the homogeneity of variance were examined. Results are shown as means with pooled standard error of the mean (SEM). Duncan’s multiple range test was utilized to determine significant differences among treatment means. Differences were considered statistically significant at *p* < 0.05.

## 3. Results

### 3.1. Growth Performance

As shown in Table 2, dietary oxidized oil had no effect on BW at 21 days. However, by day 42, BW in the OOC group was significantly lower than that in the CON, MAG, and RUT groups (*p* < 0.05). During the period of days 7 to 21, no significant differences were observed in ADG, ADFI, or FCR among the five groups (*p* > 0.05). In contrast, from day 22 to 42, the ADG in the OOC group was significantly lower compared to the CON, MAG, and RUT groups (*p* < 0.05). Additionally, the ADFI during this period (days 22–42) was significantly lower in the OOC group than in the other four groups (*p* < 0.05). Over the entire experimental period (days 7–42), the ADG of the OOC group was significantly lower than that of the CON, MAG, and RUT group (*p* < 0.01), and its ADFI was significantly lower than that of the CON, MAG, and GAA group (*p* < 0.05). No abnormal mortalities occurred throughout the entire experimental period.

### 3.2. Blood Parameters

As shown in Table 3, serum levels of ALT, AST, TP, ALB, GLU, UA, urea, LDH, LDL-C, HDL-C, and TC had no significant difference among the five groups (*p* > 0.05). However, serum TG levels in the OOC group were significantly higher than those in the MAG, RUT, and CON groups (*p* < 0.05).

### 3.3. Slaughter Performance and Organ Index

As shown in Table 4, dietary oxidized oil did not significantly affect slaughter performance parameters including carcass yield, half-eviscerated rate, eviscerated rate, thigh muscle rate, breast muscle rate, or abdominal fat rate, nor did it alter heart, thymus, or liver indices (*p* > 0.05). However, the gizzard index in the OOC group was significantly lower than that in the CON and GAA groups (*p* < 0.05). The bursa of Fabricius index in the MAG group was significantly lower than that in the OOC, RUT, and GAA groups (*p* < 0.05). The spleen index was significantly elevated in the OOC group compared to the MAG, RUT, and CON groups (*p* < 0.05). In addition, the pancreas indices were significantly reduced in both the OOC and RUT groups relative to the CON group (*p* < 0.05).

### 3.4. Antioxidant Capacity

As shown in Table 5, serum T-SOD activity was significantly decreased in the OOC group compared to the CON and MAG groups (*p* < 0.05). No significant differences were observed in T-SOD activity among the groups in the liver, jejunum, ileum, and breast muscle (*p* > 0.05). Serum GSH-Px activity showed significant reductions in the OOC and GAA groups compared to the CON, MAG, and RUT groups (*p* < 0.01). Hepatic GSH-Px activity was markedly elevated in the OOC, MAG, RUT, and GAA groups relative to CON (*p* < 0.01). The activity of GSH-Px in the breast muscle of the MAG, RUT, and GAA groups was significantly higher than that in the CON group (*p* < 0.05). Serum MDA content increased significantly in the OOC, RUT, and GAA groups compared to CON (*p* < 0.01). Hepatic MDA levels were lower in the MAG and CON groups than in the OOC group (*p* < 0.05). Jejunal MDA content was reduced in the CON and MAG groups compared to the OOC, RUT, and GAA groups (*p* < 0.05). Notably, ileal MDA content in the OOC group significantly exceeded all the other groups (*p* < 0.01). The MDA content in the breast muscle of the MAG group was significantly lower than that in the OOC, RUT, and GAA groups (*p* < 0.05).

### 3.5. Intestinal Histomorphology and Barrier Function-Related Gene Expression

As shown in Table 6, jejunal villus height (VH) was significantly reduced in the OOC group compared to the MAG and GAA groups (*p* < 0.05). Ileal VH significantly decreased in the OOC group compared to all the other groups (*p* < 0.05). Oxidized oil showed no significant effects on CD, VH/CD ratio, or BWT (*p* > 0.05). *ZO-1* expression in the jejunum of the OOC group was significantly downregulated compared to that in the MAG, RUT, and GAA groups (*p* < 0.01). Additionally, ileum *ZO-1* expression was significantly lower in the OOC group than that in the other four groups (*p* < 0.01). No significant intergroup differences were observed in *Occludin* or *Claudin-1* expression (*p* > 0.05).

### 3.6. Hepatic Lipid Metabolism and Inflammation

As shown in Table 7, oxidized oil significantly increased hepatic TG content and the expression of key lipogenic genes compared to the CON group, including *fatty acid synthase* (*FASN*) (*p* < 0.05), *acetyl-CoA carboxylase alpha* (*ACACA*) (*p* < 0.01), and *sterol regulatory element binding protein-1* (*SREBP-1*) (*p* < 0.05). Compared to the OOC group, TG content reduced in the MAG group (*p* < 0.05) while *ACACA* expression significantly decreased in the MAG and RUT groups (*p* < 0.01). Fatty acid transport-related genes, such as *apolipoprotein B* (*APOB)* and *microsomal triglyceride transfer protein* (*MTTP*), were significantly upregulated in all oxidized oil-fed groups (OOC, MAG, RUT, GAA) compared to CON (*p* < 0.05). Additionally, oxidized oil significantly increased *nuclear factor kappa B1* (*NF-κB1*) and *NF-κB2* expression (*p* < 0.01). Compared to the OOC group, magnolol, rutin, and gallic acid supplementation significantly decreased *NF-κB2* expression (*p* < 0.01), while gallic acid also decreased the expression of *NF-κB1* (*p* < 0.05). There were no significant differences in *tumor necrosis factor-alpha* (*TNFα*) and *interleukin-1 beta* (*IL-1β*) expression among the five groups (*p* > 0.05).

### 3.7. Meat Quality and Differential Metabolites in Breast Muscle

As shown in Table 8, compared with the OOC group, the breast muscle of broilers fed with magnolol, rutin, and gallic acid exhibited a higher L* value (*p* < 0.01), indicating greater lightness. However, there were no significant differences in the a*, b* values. In addition, there were no significant differences in pH_24h_, shear force, or drip loss among the groups (*p* > 0.05).

**Figure 1:**
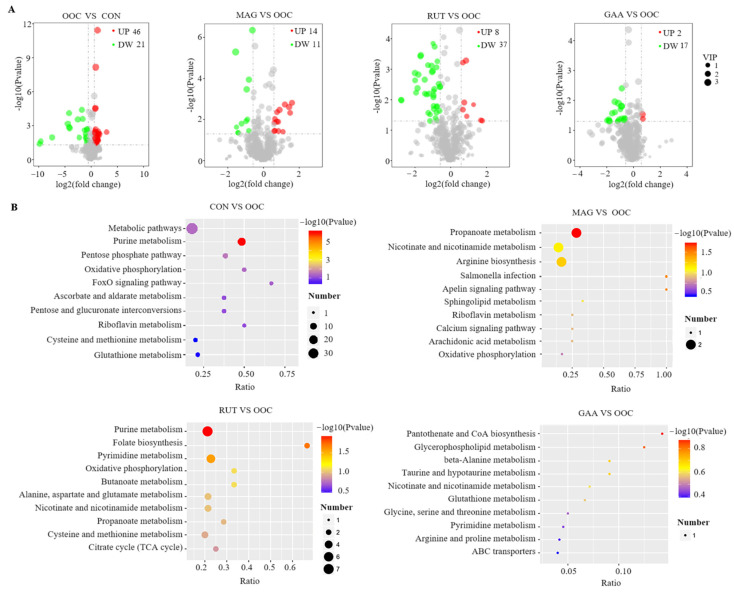
Metabolomics analysis of breast muscle in broliers (*n* = 8). (**A**) Volcano plot of differentially metabolomics. Red represents the significantly up-regulated while green represents the down-regulated. (**B**) KEGG pathway enrichment analyses of differential metabolomics.

As shown in Figure 1A, a total of 67 differential metabolites (46 up-regulated and 21 down-regulated) were identified in the OOC vs. CON comparison. These differential metabolites were significantly enriched in purine metabolism, pentose phosphate pathway, metabolic pathways, and oxidative phosphorylation (Figure 1B). Compared with the OOC group, a total of 25 (14 upregulated and 11 downregulated), 45 (8 upregulated and 37 downregulated) and 19 (2 upregulated and 17 downregulated) differential metabolites were identified in the MAG, RUT and GAA groups, respectively (Figure 1A, Tables S2–S4). Differential metabolites between the MAG and OOC groups were significantly enriched in propanoate metabolism and apelin signaling pathway. Differential metabolites between the RUT and OOC group were significantly enriched in purine metabolism, folate biosynthesis and pyrimidine metabolism (*p* < 0.05). In contrast, no enriched pathways were detected for the differentially expressed metabolites between the OOC and GAA groups.

The differential metabolites between the CON and OOC groups are listed in Table 9, which also includes the effects of magnolol, rutin or gallic acid supplementation on these differential metabolites. Among them, 14 metabolites were enriched in the purine metabolism pathway. Specifically, the levels of adenine, AMP, dGDP, ADP, dGTP, ATP, adenylyl sulfate, adenosine 5-phosphosulfate, and adenylocuccinic acid in the OOC group were significantly lower than those in the control group, while the levels of guanine, hypoxanthine, xanthosine, inosine, GMP, D-ribose-1P, and D-ribose-5P were significantly higher. Compared with the OOC group, rutin supplementation increased the level of dGDP and decreased the levels of guanine, xanthosine, and inosine. In contrast, neither magnolol nor gallic acid had a significant effect on these metabolites. Additionally, the levels of several key metabolites in the pentose phosphate pathway, including sedoheptulose-7P, D-xylulose-5P, D-ribulose-5P, D-ribose-5P, and D-ribose-1P, were significantly downregulated in the OOC group compared to the CON group (*p* < 0.05). However, supplementation with the three polyphenols did not significantly affect the levels of these metabolites compared to the OOC group. Furthermore, oxidized lipids, including 9,10-DiHOME and prostaglandin G2, were significantly elevated in the muscle of the OOC group compared to the CON group (*p* < 0.05). Notably, magnolol supplementation reduced the content of 9,10-dihome, while rutin supplementation decreased prostaglandin G2 levels (*p* < 0.05).

### 3.8. Cecal Microbiome

**Figure 2:**
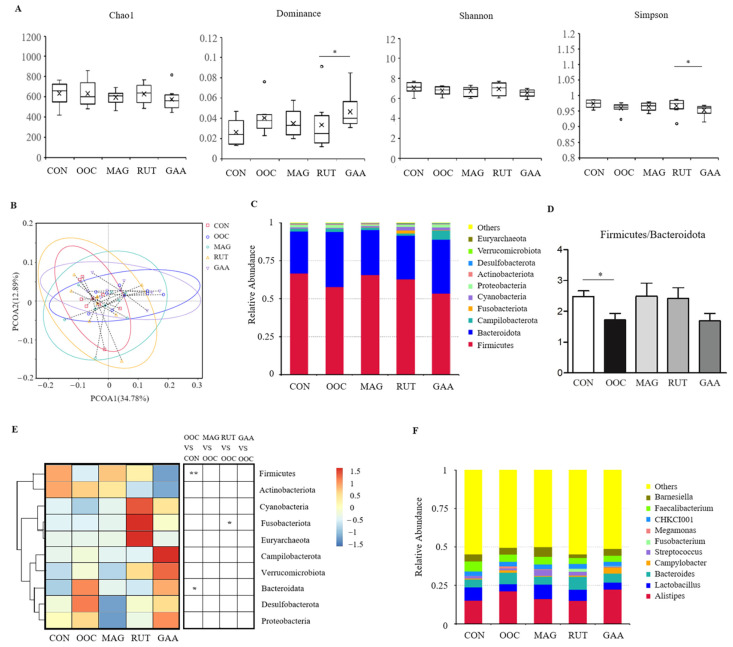
Cecal microbiota composition of briolers. (**A**) Alpha diversity of the cecal microbiota of briolers. Outliers, defined as values exceeding 1.5 times the IQR from the quartiles, are shown as open circles. (**B**) Principal coordinates analysis (PCoA) plot of cecal microbiota according to the unweighted Unifrac distance metrics. (**C**) Gut microbiota composition of brioler at the phylum level. (**D**) The ratio of Firmicutes/Bacteroidetes. (**E**) Heatmap of the differentially microbes at the phylum level. (**F**) Gut microbiota composition of brioler at the genus level. All values are expressed as the means ± SD (*n* = 8); * means *p* < 0.05; ** means *p* < 0.01.

As shown in Figure 2A, no significant differences in alpha diversity (Chao1, Dominance, Shannon, and Simpon) were found between the CON and OOC groups (*p* > 0.05). However, the Dominance index in the GAA group was significantly higher than that in the RUT group, while the Simpson indices were significantly lower (*p* < 0.05). PCoA revealed that the microbial communities across groups were not completely separated (PCoA1, 34.78%; PCoA2, 12.89%) (Figure 2B). The dominant phyla in the cecum of all groups were Firmicutes and Bacteroidetes (Figure 2C). Compared with the CON group, the OOC group showed a significant decrease in Firmicutes (*p* < 0.01) and an increase in Bacteroidetes (*p* < 0.05) (Figure 2E), resulting in a significantly reduced Firmicutes/Bacteroidetes ratio (*p* < 0.05) (Figure 2D). At the genus level, the most abundant genus was *Alistipes*, followed by *Lactobacillus* and *Bacteroides* (Figure 2F).

**Figure 3:**
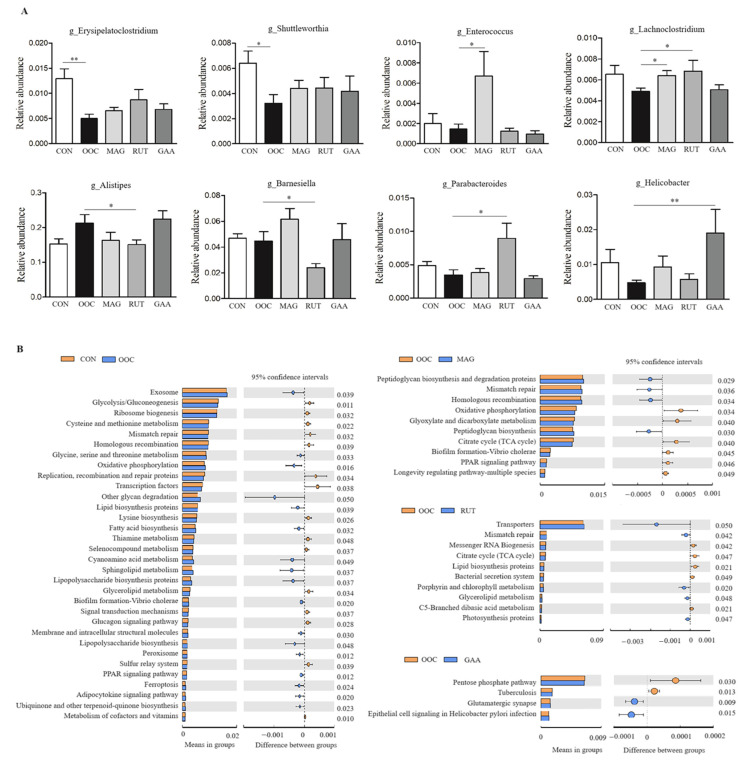
The differential genera and predictive functional profiles of the cecal microbiota of briolers. (**A**) The differential bacteria genera. (**B**) Predictive functional profiles of the ceacl microbiota of briolers. All values are expressed as the means ± SD (*n* = 8); * means *p* < 0.05; ** means *p* < 0.01.

The relative abundances of the top 50 genera are listed in Table S5, with differential abundant genera illustrated in Figure 3A. Compared to the CON group, the OOC group exhibited significantly lower abundances of *Erysipelatoclostridium* (*p* < 0.01) and *Shuttleworthia* (*p* < 0.05). Although supplementation with the three polyphenols increased the relative abundance of these genera compared to the OOC group, the differences did not reach statistical significance (*p* > 0.05). Compared to the OOC group, the abundance of *Enterococcus* was significantly increased in the MAG group, *Lachnoclostridium* was elevated in the MAG and RUT groups (*p* < 0.05), and *Helicobacter* was higher in the GAA group (*p* < 0.01). In contrast, the relative abundances of *Alistipes* and *Barnesiella* were significantly reduced in the RUT group compared to the OOC group (*p* < 0.05). The predictive functions of the cecal microbiota are shown in Figure 3B. Compared to the CON group, the OOC group exhibited decreased activity in key energy metabolism pathways (glycolysis/gluconeogenesis and glycerolipid metabolism), but showed increased activity in lipid regulatory pathways (sphingolipid metabolism and PPAR signaling) and lipopolysaccharide biosynthesis (*p* < 0.05). Compared to the OOC group, magnolol supplementation led to a reduction in pathways related to oxidative phosphorylation, glyoxylate and dicarboxylate metabolism, citrate cycle and PPAR signaling pathway. Rutin supplementation reduced activity in pathways associated with citrate cycle, lipid biosynthesis proteins, bacterial secretion system, and C5-branched dibasic acid metabolism, while increasing those related to porphyrin and chlorolipid metabolism and glycerolipid metabolism.

### 3.9. The Microbial Correlation Analyses

**Figure 4:**
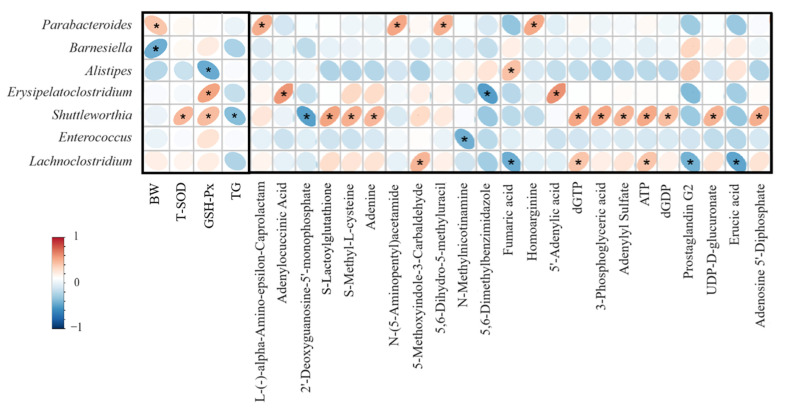
The microbial correlation analyses. Red represents positive correlation while blue represents negative correlation. * means *p* < 0.05. Abbreviations: BW: body weight, T-SOD: total superoxide dismutase, GSH-Px: glutathione peroxidase.

As shown in Figure 4, BW at 42 d was positively correlated with the abundance of *Parabacteroides* but negatively correlated with *Barnesiella*. Serum T-SOD levels showed a positive correlation with *Shuttleworthia* abundance. Serum GSH-Px activity was positively correlated with the abundance of *Erysipelatoclostridium* and *Shuttleworthia*, but negatively correlated with the abundance of *Alistipes*. Serum TG levels showed negative correlation with the abundance of *Shuttleworthia*.

Regarding muscle metabolites, the abundance of *Parabacteroides* was positively correlated with the levels of L-(-)-alpha-Amino-epsilon-Caprolactam, N-(5-Aminopentyl) acetamide, 5,6-dihydro-5-methyluracil, and hypoxanthine. The abundance of *Alistipes* was positively correlated with fumaric acid levels. *Erysipelatoclostridium* abundance showed a positive correlation with adenylosuccinic acid and 5′-adenylic acid, but a negative correlation with 5,6-dimethylbenzimidazole. *Shuttleworthia* abundance was positively correlated with the levels of S-lactoylglutathione, S-methyl-L-cysteine, adenine, dGTP, 3-phosphoglyceric acid, adenylyl sulfate, ATP, dGDP, UDP-D-glucuronate, and adenosine 5′-diphosphate, but negatively correlated with 2′-deoxyguanosine-5′-monophosphate. *Enterococcus* abundance was negatively correlated with N-methylnicotinamine. *Lachnoclostridium* abundance was positively correlated with 5-methoxyindole-3-carbaldehyde, dGTP, and ATP, but negative correlated with fumaric acid, prostaglandin G2 and erucic acid.

## 4. Discussion

Previous studies reported that broilers fed oxidized soybean oil diet exhibit lower final body weight, body weight gain, feed efficiency, and feed intake [19,20]. Consistent with these studies, our experiment demonstrated that broilers fed with oxidized oil showed a significant reduction in growth performance, specifically in BW (42 d), ADG and ADFI during days 7 to 42. This reduction may be attributed to both the unpalatable odors and flavors of oxidized oil, which decrease feed intake [21], and reactions between lipid oxidation products and dietary components (e.g., vitamins, proteins) that diminish nutritional value [1]. Additionally, organ damage such as intestinal injury may also contribute to decreased production performance. Contrastingly, Zhang et al. (2022) reported that relacing 50% of the nonoxidized oil in the dietary fat blend with oxidized corn oil had no significant effect on body weight gain, but increased the feed intake of broiler, compared to a diet containing only nonoxidized oil [16]. Chen et al. (2023) similarly noted that 4% oxidized soybean oil significantly increased feed intake on d 1 to 21 and weight gain on d 1 to 7 of broiler chicks compare with the fresh oil group [22]. These discrepancies could be attributed to variations in oxidized lipid type, concentration, or experimental models. Moreover, the adverse effects of oxidized oil on growth performance were significantly stronger in the later phase (days 22–42) compared to the earlier stage (days 7–21), indicating cumulative damage that becomes most evident during periods of peak energy demand. In our study, the supplementation of magnolol and rutin effectively mitigated the detrimental effects of oxidized oil on growth performance, specifically improving BW (42 d) and ADG on d 7–42. This finding aligns with earlier research indicating that magnolol and rutin promote growth by reducing oxidative stress and enhancing antioxidant capacity [23,24]. Furthermore, consistent with previous studies [12,24,25], all three polyphenols significantly improved ADFI, likely due to their ability to alleviate oxidative damage and promote digestive tract health.

No significant differences were observed in slaughter performance parameters, indicating limit impact of oxidized oil on overall carcass yield. However, oxidized oil can cause damage to certain organs and lead to changes in their relative weights [8]. In our study, the oxidized oil group exhibited a significantly increased spleen index alongside decreased gizzard and pancreas indices, indicates potential oxidative stress-induced damage to these organs. The reduced weight and functional impairment of the gizzard and pancreas may result from both the direct toxic effects of oxidized oil and the associated reduction in feed intake. The spleen, a key immune organ, typically undergoes enlargement in response to increased inflammation and immune activation [26]. Magnolol and rutin supplementation attenuated these changes, potentially through modulation of immune responses and mitigation of oxidative organ damage. Additionally, magnolol appears to inhibit hyperplasia of the bursa of Fabricius, which could be attributed to its superior antioxidant properties.

It is well established that long-term intake of oxidized oil can cause oxidative stress. Lipid peroxidation products such as MDA and the activities of antioxidant enzyme such as T-SOD and GSH-Px, serve as effective indicators for evaluating oxidative stress. Previous studies have shown that feeding oxidized oil increases MDA content while decreasing antioxidant enzyme activities [22,27,28]. Consistent with these findings, our study observed that oxidized oil elevated MDA levels in serum, liver, jejunum, and ileum, and decreased serum T-SOD and GSH-Px activities. Notably, oxidized oil significantly increased hepatic GSH-Px activity, suggesting that moderate oxidation triggers compensatory antioxidant responses to cope with ongoing oxidative challenges [29]. The reduced antioxidant enzyme activity in serum reflects systemic oxidative stress. Magnolol supplementation significantly improved serum T-SOD activity and reduced MDA levels in liver, intestine and breast muscle, demonstrating robust antioxidant efficacy properties consistent with our previous reports [13,30]. Notable, the MDA content in the breast muscle of the MAG group was significantly lower than that in the RUT and GAA groups. These results suggest that although all three polyphenols alleviated oxidative stress, magnolol demonstrated superior efficacy in enhancing overall antioxidant activity compared to rutin or gallic acid at the dietary supplementation level of 200 mg/kg used in this study.

Consistent with reports linking oxidized oil to intestinal barrier damage [22,31], our study demonstrated significant reductions in intestinal VH and the expression of intestinal barrier function gene *ZO-1*. VH serves as a key indicator of intestinal health, where its impairment directly reduces intestinal absorptive surface area and compromises nutrient uptake efficiency [32]. Additionally, decreased expression of intestinal barrier function genes increases intestinal permeability, elevating risks of bacterial translocation and toxin exposure. Consistent with previous reports [26,33,34], our results indicated that all three polyphenols significantly increased villus height (VH) and the expression of the tight junction protein ZO-1 in both the jejunum and ileum. The enhancement of intestinal barrier function may be one mechanism through which dietary supplementation with these polyphenols improves broiler growth performance.

It is well-established that oxidized oil disrupts hepatic lipid metabolism. Dietary oxidized oil has been shown to increase hepatic triglyceride levels in laying hens [35]. Supplementation with oxidized oil in broiler diet can increase plasma triglyceride and cholesterol concentrations [20,36]. Consistent with these studies, our study demonstrated that oxidized oil significantly increased serum triglycerides and upregulated hepatic genes involved in fatty acid synthesis (*FASN*, *ACACA*, *SREBP-1*) and lipid transport (*APOB, MTTP*). The addition of the three polyphenols significantly reduced the serum TG level. While dietary magnolol [37], rutin [38] and gallic acid [39] all positively regulate lipid metabolism, magnolol demonstrated superior efficacy in ameliorating hepatic lipid disorders in our study. In addition to lipid metabolic disorders, oxidized oils also induce liver inflammation. Oxidized oil has been shown to trigger inflammatory response [16,31]. Consistently, our results revealed that oxidized oil significantly increased the mRNA expression of *NF-κB1* and *NF-κB2*. NFκB1 plays an important role in immune and inflammatory responses while NF-κB2 is critical in the organogenesis of peripheral lymphoid tissues and B-cell development [40]. In this study, all three polyphenols significantly suppressed the expression of *NF-κB2*, while gallic acid also exhibited a suppression effect of *NF-κB1.* In summary, our findings indicate that these compounds can alleviate oxidative stress-induced inflammation by inhibiting the NF-κB signaling pathway.

Meat quality serves as a key determinant for consumers’ purchasing decisions. The meat color serves as a critical quality indicator. Oxidation of the lipid component are associated with meat deterioration and reduced lightness (L*) [41]. Our results indicated that all three polyphenols supplementation increased muscle lightness (L*), likely attributable to their mitigation of oxidative damage. However, oxidized oil and polyphenols had no significant effect on other meat redness (a*), yellowness (b*), pH24h, shear force, and drip loss. Meat metabolites significantly influence quality characteristics. The differential metabolites influenced by oxidized oil were enriched in purine metabolism, pentose phosphate pathway, and oxidative phosphorylation. Purine metabolism plays an important role in energy homoeostasis, cell survival, and proliferation [42], while simultaneously serving as a critical pathway influencing meat flavor development [43]. In our study, oxidized oil significantly downregulated energy-related purine metabolites (ATP, AMP, ADP, dGTP) while increasing purine breakdown products (hypoxanthine, xanthosine, inosine). This finding points to a severe disruption of purine metabolism and mitochondrial energy production in broilers. We speculated that reactive oxygen species derived from the oxidized oil impair the mitochondrial function, leading to inefficient oxidative phosphorylation and a consequent depletion of ATP pools. This cellular energy crisis provides a mechanistic explanation for the observed growth performance deficits in the OOC group, as ATP is indispensable for protein synthesis and muscle development [44]. All three polyphenols partially counteracted the effects of oxidized oil on key metabolites in these pathways, with rutin demonstrating superior efficacy in enhancing dGTP while decreasing guanine, xanthosine, and inosine levels. Lipid oxidation not only adversely affects the sensory and functional properties of meat but also generates free radicals and toxic compounds, which may contribute to disease development and pose potential health risks to consumers [45]. Notably, oxidized oil led to a significant accumulation of lipid oxidation products (9,10-DiHOME and prostaglandin G2). 9,10-DiHOME, a dihydroxy fatty acid derived from linoleic acid oxidation, often indicates heightened lipid peroxidation and oxidative stress [46]. In our study, the reduction of 9,10-DiHOME by magnolol implies its effectiveness in antioxidant properties and attenuating lipid peroxidation. Prostaglandin G2 is synthesized from arachidonic acid during inflammation or injury [47]. Rutin-mediated reduction in prostaglandin G2 suggests potent anti-inflammatory properties.

The gut microbiota critically influences chicken health, growth, and meat quality. High microbial diversity reflects a more stable microbiota community. Consistent with the previous reports [31], oxidized oil did not significantly alter the alpha diversity of the cecal microbiota. Notably, the α-diversity in the RUT group was higher than that observed in the GAA group. However, oxidized oil modified gut microbial composition, notably reducing the Firmicutes/Bacteroidetes ratio, which is associated with weight gain [48]. Additionally, oxidized oil significantly reduced the abundance of *Erysipelotrichaceae* and *Shuttleworthia*. *Erysipelotrichaceae* plays a role in immunometabolic regulations and the maintenance of intestinal health [49,50]. Notably, our study confirmed a positive correlation between *Erysipelotrichaceae* abundance and serum GSH-Px activity. *Shuttleworthia*, which correlates with improved gut health [51], and positively associates with BW, FI, and FCR in broilers [52], was further identified here to exhibit positive correlations with serum T-SOD and GSH-Px activities. The decrease in beneficial bacteria reflects the imbalance of intestinal flora caused by oxidized oil. Notably, oxidized oil activated the lipopolysaccharide biosynthesis pathway, which partly explains the observed inflammatory response (elevated hepatic NF-κB levels) and immune dysregulation (bursal index abnormalities) in this study. The gut microbiota contributes to meat metabolism [53]. In our study, the abundance of *Shuttleworthia* showed positive correlations with muscle dGTP, ATP, and dGDP levels. The intestinal flora disorder caused by oxidized oil may partly explain the inhibition of muscle energy metabolism.

Although the three polyphenol intervention groups all increased the relative abundance of *Erysipelotrichaceae* and *Shuttleworthia* to some extent, the differences were not statistically significant. Notably, magnolol and rutin significantly increased *Lachnoclostridium* abundance which produces short-chain fatty acids and exhibits anti-inflammatory properties [54,55]. Our study revealed *Lachnoclostridium* abundance positively correlated with dGTP, ATP but negatively correlated with rumaric acid and prostaglandin G2. Additionally, magnolol significantly increased *Enterococcus* abundance which plays a positive role in intestinal health, growth and immunity in poultry [56,57]. Rutin additionally elevated *Parabacteroides*, while reducing *Alistipes* and *Barnesiella* proportion. *Parabacteroides* is a commensal bacterium which strengthens intestinal barrier function [58] and benefits liver health [59,60], with our study confirming its positive correlation with BW. *Barnesiella* and *Alistipes*, whose reduction has been linked to attenuated mucosal damage [61], were further identified in this study to be negatively correlated with BW and GSH-Px activity, respectively. Through these microbiota-directed mechanisms, the polyphenols collectively alleviate oxidized oil-induced impairments in poultry health, growth performance, and meat quality.

## 5. Conclusions

Oxidized oil negatively affected the growth performance, antioxidant capacity, intestinal health, hepatic lipid metabolism and meat quality of broilers. Under the experimental conditions of this study, although supplementation with magnolol, rutin, and gallic acid alleviated the adverse effects induced by oxidized oil to varying degrees, magnolol and rutin were more effective than gallic acid in mitigating the negative impacts on growth performance, gut microbiota composition, and meat quality. In addition, magnolol demonstrated superior efficacy in enhancing antioxidant capacity. It should be noted that this study has several limitations. For instance, the research utilized an animal model of only a single strain and gender. Additionally, the specific mechanisms underlying the observed changes in the gut microbiota and metabolome remain to be further explored. Nonetheless, the findings of this study provide valuable references for the application of these polyphenols as functional feed additives in poultry production.

## Figures and Tables

**Table 1:** Ingredients and nutrient composition of the basal diet fed to broilers.

Ingredients (%)	7–21 d	22–42 d	Nutrient Levels ^2^	7–21 d	22–42 d
Maize	55.0	61.8	Metabolizable energy (MJ/kg)	12.54	12.84
Soybean meal	36.8	30.6	Crude protein (%)	21.55	19.20
Soybean oil	4.0	4.0	Methionine (%)	0.52	0.44
Dicalcium phosphate	1.8	1.5	Lysine (%)	1.42	1.13
Limestone	1.0	0.9	Calcium (%)	1.02	0.88
Sodium chloride	0.3	0.3	Total phosphorus (%)	0.76	0.70
Choline chloride (50%)	0.3	0.3			
DL-methionine (98%)	0.2	0.15			
L-lysine (78%)	0.3	0.15			
Premix ^1^	0.3	0.3			

^1^ The premix provided the following (per kilogram of diet): vitamin A, 12,500 IU; vitamin D3, 2500 IU; vitamin K3, 2.65 mg; vitamin B1, 2 mg; vitamin B2, 6 mg; vitamin B12, 0.025 mg; vitamin E, 30 IU; biotin, 0.0325 mg; folic acid, 1.25 mg; pantothenic acid, 12 mg; niacin, 50 mg; manganese, 100 mg; zinc, 75 mg; iron, 80 mg; copper, 8 mg; selenium, 0.25 mg; iodine, 0.35 mg. ^2^ Metabolizable energy was calculated using data provided by the Feed Database in China (2020) while other nutrient levels were measured values.

**Table 2:** Effects of dietary magnolol, rutin, and gallic acid on growth performance of broilers fed oxidized oil.

Items	CON	OOC	MAG	RUT	GAA	SEM	*p*
BW at 21 d (g)	591.58	603.85	609.17	598.96	605.52	3.92	0.686
BW at 42 d (g)	2118.03 ^a^	1961.71 ^b^	2077.85 ^a^	2084.94 ^a^	2049.77 ^ab^	16.87	0.031
7–21 d							
ADG (g/day)	31.33	32.34	32.78	31.93	32.30	0.29	0.603
ADFI (g/day)	33.95	35.44	34.69	34.32	34.70	0.21	0.227
FCR (g/g)	1.63	1.63	1.59	1.62	1.61	0.01	0.540
22–42 d							
ADG (g/day)	72.69 ^a^	64.66 ^b^	69.94 ^a^	70.76 ^a^	68.77 ^ab^	0.82	0.019
ADFI (g/day)	132.24 ^a^	121.30 ^b^	131.48 ^a^	128.03 ^a^	128.75 ^a^	1.16	0.016
FCR (g/g)	1.91	2.01	1.95	1.93	1.99	0.02	0.282
7–42 d							
ADG (g/day)	54.32 ^a^	49.29 ^c^	53.64 ^ab^	52.58 ^ab^	51.70 ^bc^	0.47	0.003
ADFI (g/day)	92.92 ^a^	86.96 ^b^	92.76 ^a^	90.54 ^ab^	91.13 ^a^	0.68	0.027
FCR (g/g)	1.71	1.77	1.73	1.72	1.76	0.01	0.511

Abbreviations: CON = fresh oil group; OOC = oxidized oil group; MAG = oxidized oil plus 200 mg/kg of magnolol; RUT = oxidized oil plus 200 mg/kg of rutin; GAA = oxidized oil plus 200 mg/kg of gallic acid; BW = body weight; ADG = average daily gain; ADFI = average daily feed intake; FCR = feed conversion ratio; SEM = pooled standard error of the mean. ^a–c^ Values within a row with different superscripts differ significantly at *p* < 0.05. All values are expressed as the means (*n* = 8).

**Table 3:** Effects of dietary magnolol, rutin, and gallic acid on blood parameters of broilers fed oxidized oil.

Items	CON	OOC	MAG	RUT	GAA	SEM	*p*
ALT (U/L)	4.77	6.31	5.51	5.40	4.98	0.30	0.545
AST (U/L)	327.24	320.50	360.06	268.28	321.15	13.92	0.397
TP (g/L)	28.81	27.99	30.90	28.60	29.52	0.73	0.826
ALB (g/L)	12.90	11.87	13.36	12.71	12.60	0.26	0.559
GLU (mM/L)	8.96	9.97	7.34	9.30	9.30	0.38	0.347
UA (mM/L)	0.38	0.26	0.24	0.28	0.26	0.02	0.155
urea (mM/L)	0.61	0.58	0.53	0.51	0.52	0.02	0.158
LDH (U/L)	1797.26	1690.52	1969.55	1619.00	1759.09	76.09	0.742
HDL-C (mM/L)	2.46	2.54	2.36	2.60	2.56	0.04	0.503
LDL-C (mM/L)	0.48	0.66	0.60	0.62	0.55	0.02	0.065
TC (mM/L)	3.02	3.26	3.04	3.33	3.28	0.06	0.311
TG (mM/L)	0.36 ^b^	0.46 ^a^	0.37 ^b^	0.37 ^b^	0.39 ^b^	0.01	0.020

Abbreviations: CON = fresh oil group; OOC = oxidized oil group; MAG = oxidized oil plus 200 mg/kg of magnolol; RUT = oxidized oil plus 200 mg/kg of rutin; GAA = oxidized oil plus 200 mg/kg of gallic acid; SEM = pooled standard error of the mean; AST = aspartate amino transferase; ALT = alanine aminotransferase; TP = total protein; ALB = albumin; GLU = glucose; UA = uric acid; LDH = lactate dehydrogenase; HDL-C = high-density lipoprotein cholesterol; LDL-C = low-density lipoprotein cholesterol; TC = total cholesterol; TG = triglyceride. ^a, b^ Values within a row with different superscripts differ significantly at *p* < 0.05. All values are expressed as the means (*n* = 8).

**Table 4:** Effects of dietary magnolol, rutin, and gallic acid on slaughter performance of broilers fed oxidized oil.

Items (%)	CON	OOC	MAG	RUT	GAA	SEM	*p*
Carcass yield	92.57	92.31	93.36	93.01	92.90	0.27	0.796
Half-eviscerated rate	84.69	85.69	85.76	85.59	85.21	0.30	0.786
Eviscerated rate	72.12	73.41	73.97	73.37	71.97	0.33	0.237
Thigh muscle rate	18.60	18.74	19.81	19.08	18.66	0.25	0.564
Breast muscle rate	24.10	24.10	26.57	24.40	23.44	0.60	0.585
Abdominal fat rate	2.13	1.86	1.74	1.91	1.92	0.09	0.759
Gizzard	1.64 ^a^	1.39 ^b^	1.51 ^ab^	1.50 ^ab^	1.59 ^a^	0.03	0.038
Heart	0.43	0.45	0.42	0.43	0.41	0.01	0.646
Bursa of Fabricius	0.17 ^ab^	0.21 ^a^	0.14 ^b^	0.21 ^a^	0.22 ^a^	0.01	0.048
Thymus	0.49	0.46	0.42	0.48	0.46	0.02	0.857
Liver	1.84	1.80	1.91	1.93	1.83	0.03	0.711
Spleen	0.09 ^b^	0.17 ^a^	0.10 ^b^	0.11 ^b^	0.12 ^ab^	0.01	0.046
Pancreas	0.23 ^a^	0.20 ^b^	0.21 ^ab^	0.20 ^b^	0.22 ^ab^	0.07	0.022

CON = fresh oil group; OOC = oxidized oil group; MAG = oxidized oil plus 200 mg/kg of magnolol; RUT = oxidized oil plus 200 mg/kg of rutin; GAA = oxidized oil plus 200 mg/kg of gallic acid; SEM = pooled standard error of the mean. ^a, b^ Values within a row with different superscripts differ significantly at *p* < 0.05. All values are expressed as the means (*n* = 8).

**Table 5:** Effects of dietary magnolol, rutin, and gallic acid on antioxidant capacity of broilers fed oxidized oil.

	Items	CON	OOC	MAG	RUT	GAA	SEM	*p*
Serum	T-SOD (U/mL)	338.39 ^a^	250.30 ^b^	335.93 ^a^	287.48 ^ab^	310.22 ^ab^	15.11	0.028
	GSH-Px (U/mL)	6.69 ^a^	5.70 ^b^	6.48 ^a^	6.08 ^a^	5.75 ^b^	0.07	<0.001
	MDA (nM/mL)	3.80 ^b^	5.98 ^a^	4.62 ^ab^	6.06 ^a^	5.36 ^a^	0.26	0.020
Liver	T-SOD (U/mgprot)	637.05	638.36	603.32	614.45	632.41	8.26	0.618
	GSH-Px (U/mgprot)	23.48 ^b^	35.3 ^a^	35.7 ^a^	36.93 ^a^	34.84 ^a^	1.25	<0.001
	MDA (nM/mgprot)	3.07 ^b^	4.04 ^a^	3.32 ^b^	3.71 ^ab^	3.79 ^ab^	0.11	0.017
Jejunum	T-SOD (U/mgprot)	317.96	345.10	329.51	350.71	345.89	7.61	0.659
	GSH-Px (U/mgprot)	9.53	11.26	9.80	7.74	10.93	0.53	0.248
	MDA (nM/mgprot)	1.13 ^b^	1.81 ^a^	1.21 ^b^	1.75 ^a^	1.81 ^a^	0.09	0.015
Ileum	T-SOD (U/mgprot)	183.67	210.62	182.16	189.44	171.76	5.13	0.174
	GSH-Px (U/mgprot)	5.61	4.14	4.2	5.32	5.66	0.28	0.210
	MDA (nM/mgprot)	0.61 ^b^	0.92 ^a^	0.57 ^b^	0.71 ^b^	0.67 ^b^	0.03	0.002
Breast muscle	T-SOD (U/mgprot)	103.41	101.51	99.83	100.70	95.95	2.87	0.955
	GSH-Px (U/mgprot)	3.99 ^b^	4.22 ^ab^	4.64 ^a^	4.50 ^a^	4.69 ^a^	0.08	0.018
	MDA (nM/mgprot)	0.14 ^bc^	0.18 ^ab^	0.12 ^c^	0.18 ^a^	0.17 ^ab^	0.01	0.022

Abbreviations: CON = fresh oil group; OOC = oxidized oil group; MAG = oxidized oil plus 200 mg/kg of magnolol; RUT = oxidized oil plus 200 mg/kg of rutin; GAA = oxidized oil plus 200 mg/kg of gallic acid; SEM = pooled standard error of the mean; T-SOD, total superoxide dismutase; GSH-Px, glutathione peroxidase; MDA, malondialdehyde. ^a–c^ Values within a row with different superscripts differ significantly at *p* < 0.05. All values are expressed as the means (*n* = 8).

**Table 6:** Effects of dietary magnolol, rutin, and gallic acid on intestinal histomorphology and barrier function-related gene expression of broilers fed oxidized oil.

	Items	CON	OOC	MAG	RUT	GAA	SEM	*p*
Jejunum	VH (μm)	1551.98 ^ab^	1468.19 ^b^	1725.85 ^a^	1594.89 ^ab^	1705.63 ^a^	31.84	0.044
	CD (μm)	161.90	157.19	178.78	159.69	185.02	10.94	0.531
	VH/CD	10.11	10.17	10.34	10.25	10.12	0.28	0.999
	BWT (μm)	247.01	215.49	236.80	221.87	257.94	8.29	0.492
	*ZO-1*	1.01 ^bc^	0.79^c^	1.23 ^ab^	1.26 ^a^	1.06 ^ab^	0.05	0.005
	*Occludin*	1.03	1.02	1.23	1.24	1.31	0.08	0.769
	*Claudin1*	1.01	0.76	0.94	1.31	0.85	0.05	0.130
Ileum	VH (μm)	1075.95 ^a^	949.88 ^b^	1168.75 ^a^	1082.27 ^a^	1045.48 ^a^	18.79	0.002
	CD (μm)	157.63	153.83	175.97	160.72	161.18	4.50	0.621
	VH/CD	6.92	6.10	6.97	6.87	6.50	0.18	0.497
	BWT (μm)	208.93	210.37	198.05	203.83	226.37	6.77	0.770
	*ZO-1*	1.02 ^a^	0.40 ^b^	1.10 ^a^	0.97 ^a^	0.87 ^a^	0.07	0.002
	*Occludin*	1.05	1.12	1.39	1.16	1.47	0.08	0.441
	*Claudin1*	1.02	1.09	1.02	0.86	1.00	0.06	0.817

Abbreviations: CON = fresh oil group; OOC = oxidized oil group; MAG = oxidized oil plus 200 mg/kg of magnolol; RUT = oxidized oil plus 200 mg/kg of rutin; GAA = oxidized oil plus 200 mg/kg of gallic acid; SEM = pooled standard error of the mean; VH = villus height; CD = crypt depth; BWT = bowel wall thickness; *ZO-1* = Zonula Occludens-1. ^a–c^ Values within a row with different superscripts differ significantly at *p* < 0.05. All values are expressed as the means (*n* = 8).

**Table 7:** Effects of dietary magnolol, rutin, and gallic acid on hepatic lipid metabolism and inflammation of broilers fed oxidized oil.

	CON	OOC	MAG	RUT	GAA	SEM	*p*
TG (mM/gprot)	0.20 ^c^	0.28 ^a^	0.22 ^bc^	0.25 ^abc^	0.27 ^ab^	0.01	0.031
*FASN*	1.02 ^b^	2.23 ^a^	2.67 ^a^	2.10 ^a^	2.44 ^a^	0.16	0.017
*ACACA*	1.08 ^b^	6.36 ^a^	1.98 ^b^	2.43 ^b^	6.77 ^a^	0.69	0.008
*SREBP-1*	1.04 ^c^	2.45 ^a^	2.88 ^a^	2.07 ^ab^	1.92 ^abc^	0.18	0.015
*PPARα*	1.05	1.05	1.26	1.06	1.12	0.08	0.914
*APOB*	1.05 ^b^	3.77 ^a^	2.83 ^a^	3.11 ^a^	3.25 ^a^	0.26	0.004
*MTTP*	1.05 ^b^	2.67 ^a^	2.20 ^a^	2.14 ^a^	2.36 ^a^	0.18	0.038
*NF-κB1*	1.01 ^c^	1.67 ^a^	1.52 ^ab^	1.47 ^ab^	1.24 ^bc^	0.07	0.006
*NF-κB2*	1.05 ^c^	3.20 ^a^	1.92 ^b^	2.16 ^b^	2.45 ^b^	0.17	0.001
*TNF-α*	1.11	1.29	1.32	1.18	1.15	0.06	0.841
*IL-1β*	1.07	1.06	1.23	1.09	1.65	0.18	0.829

Abbreviations: CON = fresh oil group; OOC = oxidized oil group; MAG = oxidized oil plus 200 mg/kg of magnolol; RUT = oxidized oil plus 200 mg/kg of rutin; GAA = oxidized oil plus 200 mg/kg of gallic acid; SEM = pooled standard error of the mean; TG = triglycerides; FASN = fatty acid synthase; ACACA = acetyl-CoA carboxylase alpha; SREBP-1 = sterol regulatory element binding protein-1; PPARα = peroxisome proliferator-activated receptor alpha; APOB = apolipoprotein; MTTP = microsomal triglyceride transfer protein; NF-κB = nuclear factor kappa B; TNF-α = tumor necrosis factor-alpha; IL-1β = interleukin-1 beta. ^a–c^ Values within a row with different superscripts differ significantly at *p* < 0.05. All values are expressed as the means (*n* = 8).

**Table 8:** Effects of dietary magnolol, rutin, and gallic acid on meat quality of broilers fed oxidized oil.

	CON	OOC	MAG	RUT	GAA	SEM	*p*
L*	53.12 ^ab^	51.14 ^b^	54.85 ^a^	54.08 ^a^	54.61 ^a^	0.39	0.009
a*	3.94	3.94	3.91	3.68	4.99	0.23	0.473
b*	10.77	11.28	10.69	10.42	11.67	0.27	0.655
pH_24h_	5.79	5.78	5.80	5.78	5.74	0.05	0.783
Shear force (N/cm^2^)	3.79	3.32	3.41	3.92	4.01	0.14	0.422
drip loss (%)	7.06	8.49	6.33	6.6	6.56	0.38	0.408

Abbreviations: CON = fresh oil group; OOC = oxidized oil group; MAG = oxidized oil plus 200 mg/kg of magnolol; RUT = oxidized oil plus 200 mg/kg of rutin; GAA = oxidized oil plus 200 mg/kg of gallic acid; SEM = pooled standard error of the mean. ^a, b^ Values within a row with different superscripts differ significantly at *p* < 0.05. All values are expressed as the means (*n* = 8).

**Table 9:** Effects of dietary magnolol, rutin, and gallic acid on meat metabolomic profile of broilers fed oxidized oil (*n* = 8).

Metabolite	Formula	OOC vs. CON		MAG vs. OOC		RUT vs. OOC		GAA vs. OOC	
		FC	*p*	FC	*p*	FC	*p*	FC	*p*
S-Methyl-L-cysteine	C_4_H_9_NO_2_S	0.28	<0.001	0.74	0.123	0.78	0.201	0.87	0.516
Nicotinuric Acid	C_8_H_8_N_2_O_3_	0.56	0.002	1.56	0.035	1.69	0.012	1.48	0.022
L-Leucyl-L-Alanine	C_9_H_18_N_2_O_3_	2.61	0.003	0.43	0.016	0.43	0.006	1.06	0.593
N-Acetylhistamine	C_7_H_11_N_3_O	2.21	0.008	0.63	0.187	0.50	<0.001	0.78	0.087
S-Lactoylglutathione	C_13_H_21_N_3_O_8_S	0.005	0.011	1.05	0.801	1.30	0.184	7.14	0.216
Glutathione	C_10_H_17_N_3_O_6_S	1.58	0.012	0.90	0.575	0.65	0.023	0.75	0.100
L-(-)-alpha-Amino-epsilon-Caprolactam	C_6_H_12_N_2_O	0.55	0.019	1.41	0.193	1.53	0.138	0.91	0.982
Val-Ser	C_8_H_16_N_2_O_4_	2.03	0.019	0.54	0.051	0.56	0.040	0.74	0.287
S-Adenosyl-L-homocysteine	C_14_H_20_N_6_O_5_S	1.63	0.026	0.71	0.101	0.48	0.001	0.69	0.065
Glycyl-L-leucine	C_8_H_16_N_2_O_3_	1.86	0.036	0.84	0.709	0.79	0.577	0.53	0.094
Phe-Pro	C_14_H_18_N_2_O_3_	1.84	0.041	1.08	0.671	0.83	0.701	0.89	0.871
Phosphocholine	C_5_H_15_NO_4_P^+^	2.17	0.005	0.56	0.035	0.51	0.016	0.46	0.012
N-(5-Aminopentyl) acetamide	C_7_H_16_N_2_O	0.44	0.012	3.02	0.002	3.23	0.047	0.98	0.884
Adenine	C_5_H_5_N_5_	0.32	<0.001	0.67	0.091	0.68	0.098	0.80	0.360
AMP	C_10_H_14_N_5_O_7_P	0.17	<0.001	0.81	0.853	1.23	0.340	0.91	0.863
dGDP	C_10_H_15_N_5_O_10_P_2_	0.044	0.001	1.44	0.121	2.43	0.015	1.61	0.354
ADP	C_10_H_15_N_5_O_10_P_2_	0.052	0.001	1.41	0.142	2.16	0.069	1.65	0.339
Adenylyl Sulfate	C_10_H_14_N_5_O_10_PS	0.056	0.002	1.25	0.238	2.04	0.068	1.71	0.233
NADP^+^	C_21_H_28_N_7_O_17_P_3_	0.40	0.002	0.80	0.283	1.24	0.566	0.95	0.773
5′-Deoxy-5′-(methylthio)adenosine	C_11_H_15_N_5_O_3_S	1.57	0.003	0.70	0.019	0.66	0.004	0.86	0.222
beta-Nicotinamide mononucleotide	C_11_H_15_N_2_O_8_P	7.81	0.004	0.29	0.089	0.76	0.284	0.26	0.047
S-Methyl-5′-thioadenosine	C_11_H_15_N_5_O_3_S	1.57	0.004	0.68	0.012	0.66	0.004	0.84	0.156
Uridine	C_9_H_12_N_2_O_6_	2.61	0.005	0.59	0.090	0.36	0.002	0.63	0.136
Adenosine 5-phosphosulfate	C_10_H_14_N_5_O_10_PS	0.45	0.005	0.93	0.516	0.84	0.436	1.09	0.819
Guanine	C_5_H_5_N_5_O	3.13	0.005	0.59	0.167	0.25	0.001	0.51	0.103
Uracil	C_4_H_4_N_2_O_2_	1.92	0.009	0.71	0.160	0.62	0.053	0.55	0.016
Hypoxanthine	C_5_H_4_N_4_O	1.68	0.013	0.95	0.533	0.88	0.224	0.86	0.135
Cytarabine	C_9_H_13_N_3_O_5_	1.88	0.014	0.81	0.366	0.51	0.008	0.69	0.136
Tetraethylammonium fluoride dihydrate	C_8_H_24_FNO_2_	1.80	0.016	0.81	0.336	0.51	0.007	0.68	0.108
Xanthosine	C_10_H_12_N_4_O_6_	2.43	0.019	0.61	0.202	0.42	0.027	0.53	0.106
dGTP	C_10_H_16_N_5_O_13_P_3_	0.001	0.024	1.14	0.869	2.02	0.321	5.41	0.238
2′-Deoxyguanosine-5′-monophosphate	C_10_H_14_N_5_O_7_P	1.77	0.030	0.95	0.709	0.98	0.825	0.78	0.150
UDP-D-glucuronate	C_15_H_22_N_2_O_18_P_2_	0.54	0.031	1.06	0.838	0.91	0.960	0.88	0.983
Inosine	C_10_H_12_N_4_O_5_	1.88	0.035	0.72	0.156	0.41	0.001	0.69	0.107
orotidine-5P	C_10_H_13_N_2_O_11_P	1.64	0.038	0.81	0.152	0.60	0.001	0.82	0.092
ATP	C_10_H_16_N_5_O_13_P_3_	0.001	0.040	1.21	0.650	1.63	0.885	3.88	0.352
GMP	C_10_H_14_N_5_O_8_P	2.13	0.040	1.06	0.587	0.92	0.776	0.79	0.229
Cytidine	C_9_H_13_N_3_O_5_	1.77	0.040	0.77	0.371	0.52	0.020	0.74	0.313
Lycopsamine	C_15_H_25_NO_5_	1.54	0.031	0.69	0.047	0.72	0.037	0.87	0.311
Dopamine	C_8_H_11_NO_2_	2.02	0.045	0.54	0.101	0.57	0.127	0.53	0.080
Arabitol	C_5_H_12_O_5_	1.84	0.004	0.52	0.000	0.67	0.002	0.67	0.003
D-Ribulose-5P	C_5_H_11_O_8_P	2.20	0.008	1.09	0.998	0.84	0.531	0.72	0.229
3-Phosphoglyceric acid	C_3_H_7_O_7_P	0.20	0.011	0.84	0.776	0.85	0.670	1.58	0.806
Sedoheptulose-7P	C_7_H_13_BaO_10_P	2.02	0.011	1.17	0.252	0.72	0.245	0.80	0.505
D-Arabinose-5P	C_5_H_11_O_8_P	2.31	0.013	1.26	0.438	0.93	0.982	0.83	0.629
D-Xylulose-5P	C_5_H_11_O_8_P	2.19	0.017	1.08	0.726	0.80	0.553	0.72	0.347
D-Ribose-1P	C_5_H_11_O_8_P	2.21	0.022	1.15	0.521	0.76	0.536	0.91	0.773
L-Ascorbate	C_6_H_8_O_6_	2.11	0.010	0.73	0.092	0.69	0.053	0.82	0.188
Benzoic Acid	C_7_H_6_O_2_	1.72	<0.001	0.69	<0.001	0.71	0.003	0.82	0.083
4-Aminobenzoate	C_7_H_7_NO_2_	2.34	0.016	0.59	0.081	0.31	<0.001	0.66	0.146
O-Anisic Acid	C_8_H_8_O_3_	2.66	0.021	0.52	0.111	0.28	0.004	0.59	0.264
Adenylosuccinic Acid	C_14_H_18_N_5_O_11_P	0.051	<0.001	0.52	0.746	0.75	0.886	1.16	0.990
Fumaric acid	C_4_H_4_O_4_	1.86	0.006	0.81	0.305	0.53	0.005	0.85	0.464
D-Ribose-5P	C_5_H_11_O_8_P	2.56	0.009	1.13	0.577	0.86	0.783	0.84	0.709
5,6-Dimethylbenzimidazole	C_9_H_10_N_2_	1.82	<0.001	0.65	<0.001	0.76	<0.001	0.78	<0.001
N-Methylnicotinamine	C_7_H_8_N_2_O	1.52	0.008	0.72	0.187	0.82	0.194	0.92	0.673
6-Aminonicotinamide	C_6_H_7_N_3_O	1.98	0.031	0.60	0.116	0.60	0.112	0.66	0.243
Pyrrole-2-carboxylate	C_5_H_5_NO_2_	1.79	0.002	1.16	0.310	0.81	0.135	0.88	0.373
5,6-Dihydro-5-methyluracil	C_5_H_8_N_2_O_2_	0.62	0.018	1.36	0.185	1.40	0.142	0.89	0.830
3-Methylxanthine	C_6_H_6_N_4_O_2_	1.53	0.007	1.05	0.670	0.84	0.257	0.95	0.800
5-Methoxyindole-3-Carbaldehyde	C_10_H_9_NO_2_	0.57	0.002	1.53	0.014	1.39	0.083	1.09	0.551
LysoPC 10:0	C_18_H_38_NO_7_P	0.49	0.009	2.70	0.002	1.03	0.717	0.91	0.864
Ergosta-5,7,9(11),22-Tetraen-3-beta-Ol	C_28_H_42_O	0.66	0.027	1.56	0.009	1.81	0.001	1.31	0.458
9,10-DiHOME	C_18_H_34_O_4_	1.67	<0.001	0.80	0.028	1.02	0.845	0.85	0.071
Prostaglandin G2	C_20_H_32_O_6_	1.85	0.016	0.79	0.110	0.58	0.001	0.80	0.082
Erucic acid	C_22_H_42_O_2_	1.66	0.034	0.70	0.051	0.54	<0.001	0.76	0.042
2-Methylbutyroylcarnitine	C_12_H_23_NO_4_	2.05	0.047	0.65	0.223	1.00	0.457	0.50	0.057

Abbreviations: CON = fresh oil group; OOC = oxidized oil group; MAG = oxidized oil plus 200 mg/kg of magnolol; RUT = oxidized oil plus 200 mg/kg of rutin; GAA = oxidized oil plus 200 mg/kg of gallic acid; FC = fold change.

## Data Availability

The datasets of 16S rDNA gene sequencing can be found in the Sequence Read Archive database (Bioproject ID: PRJNA1182529). The original contributions presented in the study are included in the article; further inquiries can be directed to the corresponding author.

## Supplementary Materials

The following supporting information can be downloaded at https://www.mdpi.com/article/10.3390/antiox14101186/s1. Table S1. Primer sequences. Table S2. Differential metabolites in breast muscle of broilers (MAG vs. OOC groups). Table S3. Differential metabolites in breast muscle of broilers (RUT vs. OOC groups). Table S4. Differential metabolites in breast muscle of broilers (GAA vs. OOC groups). Table S5. Relative abundance of the top 50 genera in the cecum of broilers.

## Abbreviations

ACACA, acetyl-CoA carboxylase alpha; ADG, average daily gain; ADFI, average daily feed intake; ALB, albumin; ALT, alanine aminotransferase; ApoB, apolipoprotein; AST, aspartate amino transferase; BW, body weight; BWT, bowel wall thickness; CD, crypt depth; FCR, feed conversion ratio; GLU, glucose; FASN, fatty acid synthase; GSH-Px, glutathione peroxidase; HDL-C, high-density lipoprotein cholesterol; LDH, lactate dehydrogenase; IL-1β, interleukin-1 beta; LDL-C, low-density lipoprotein cholesterol; MDA, malondialdehyde; MTTP, microsomal triglyceride transfer protein; NFκB, nuclear factor kappa B; PPARα, peroxisome proliferator-activated receptor alpha; SREBP-1, sterol regulatory element binding protein-1; TC, total cholesterol; TG, triglycerides; TNF-α, tumor necrosis factor-alpha; TP, total protein; TG, triglyceride. T-SOD, total superoxide dismutase; UA, uric acid; VH, villus height.
